# Longitudinal strain analysis for assessment of early cardiotoxicity during anthracycline treatment in childhood sarcoma: A single center experience

**DOI:** 10.1002/cnr2.1852

**Published:** 2023-06-24

**Authors:** Maria Sjöborg Alpman, Annica Jarting, Kerstin Magnusson, Aristomenis Manouras, Jan‐Inge Henter, Agneta Månsson Broberg, Nikolas Herold

**Affiliations:** ^1^ Pediatric Cardiology, Astrid Lindgren Children's Hospital Karolinska University Hospital Stockholm Sweden; ^2^ Pediatric Oncology, Department of Women's and Children's Health Karolinska Institutet Stockholm Sweden; ^3^ Department of Cardiology Karolinska University Hospital Stockholm Sweden; ^4^ Department of Medicine Karolinska Institutet Stockholm Sweden; ^5^ Pediatric Oncology, Astrid Lindgren Children's Hospital Karolinska University Hospital Stockholm Sweden

**Keywords:** anthracyclines, cardiotoxicity, childhood cancer, echocardiography, strain

## Abstract

**Background:**

The growing population of long‐term childhood cancer survivors encounter a substantial burden of cardiovascular complications. The highest risk of cardiovascular complications is associated with exposure to anthracyclines and chest radiation. Longitudinal cardiovascular surveillance is recommended for childhood cancer patients; however, the optimal methods and timing are yet to be elucidated.

**Aims:**

We aimed to investigate the feasibility of different echocardiographic methods to evaluate left ventricular systolic function in retrospective datasets, including left ventricular ejection fraction (LVEF), fractional shortening (FS), global longitudinal strain (GLS) and longitudinal strain (LS) as well as the incidence and timing of subclinical left ventricular dysfunction detected by these methods.

**Methods and Results:**

A retrospective longitudinal study was performed with re‐analysis of longitudinal echocardiographic data, acquired during treatment and early follow‐up, including 41 pediatric sarcoma patients, aged 2.1–17.8 years at diagnosis, treated at Astrid Lindgren Children's Hospital, Stockholm, Sweden, during the period 2010–2021. All patients had received treatment according to protocols including high cumulative doxorubicin equivalent doses (≥250 mg/m^2^). In 68% of all 366 echocardiograms, LS analysis was feasible. Impaired LS values (<17%) was demonstrated in >40%, with concomitant impairment of either LVEF or FS in 20% and combined impairment of both LVEF and FS in <10%. Importantly, there were no cases of abnormal LVEF and FS without concomitant LS impairment.

**Conclusion:**

Our findings demonstrate feasibility of LS in a majority of echocardiograms and a high incidence of impaired LS during anthracycline treatment for childhood sarcoma. We propose inclusion of LS in pediatric echocardiographic surveillance protocols.

## 1 INTRODUCTION

In developed countries, the 5‐year overall survival after childhood cancer is approaching 85%, and the majority of children diagnosed with cancer today will be long‐term survivors.^1^ Long‐term childhood cancer survivors are at risk of treatment‐related cardiovascular complications, associated with increased morbidity and mortality.^2^, ^3^, ^4^ After high anthracycline doses during childhood (cumulative doxorubicin‐equivalent doses^5^ of ≥250 mg/m^2^), the 30‐year prevalence of symptomatic heart failure is estimated to be almost 10%, which is comparable to the prevalence of heart failure in patients aged 75–84 years in the general population.^3^, ^6^, ^7^ If radiotherapy involving the heart is added, the cumulative prevalence of heart failure rises to over 25%.^3^ The overall risk of cardiovascular complications after anthracycline treatment is dose‐dependent, with the highest risk in children treated with cumulative doxorubicin‐equivalent^5^ doses of ≥250 mg/m^2^, isolated chest radiotherapy ≥35 Gy, or with cumulative doxorubicin‐equivalent doses ≥100 mg/m2 and chest radiotherapy ≥15 Gy, if combined together.^5^, ^8^, ^9^, ^10^ Cardiovascular surveillance including echocardiography is recommended after anthracycline therapy and the frequency of surveillance is stratified by cumulative anthracycline doses.^8^, ^9^, ^10^, ^11^ However, there is a substantial variation in individual vulnerability^5^, ^12^, ^13^ with no clear cut‐off regarding safe doses of anthracyclines.^14^, ^15^ The aim of cardiac surveillance with echocardiography, during and after anthracycline therapy, is early detection of cardiac dysfunction since the prognosis is poor once clinical symptoms have evolved.^16^ International cardiovascular surveillance guidelines address long‐term follow‐up, but there is no consensus regarding surveillance during and short after anthracycline treatment.^8^, ^9^, ^10^

In children, standard methods to evaluate cardiac function by echocardiography include left ventricular ejection fraction (LVEF) and fractional shortening (FS).^17^, ^18^ Both LVEF and FS exhibit methodological limitations, including poor sensitivity for detection of early changes in cardiac function.^19^, ^20^ A significant decrease in LVEF, defined as a decrease from baseline of more than 10% to LVEF <53%, adds prognostic information regarding subsequent incidence of chronic heart failure both in children and adults during and following chemotherapy.^8^, ^9^, ^10^, ^21^, ^22^ However, cardiotoxicity is commonly defined by abnormal FS (<0.28) in pediatric cancer treatment protocols.^8^

Analysis of myocardial deformation that is, longitudinal strain (LS) or preferably global longitudinal strain (GLS), detect early signs of systolic myocardial dysfunction and are of prognostic value in adults during and after anthracycline treatment.^11^, ^22^, ^23^, ^24^, ^25^ Impairment in GLS of >15% from baseline or deformation measured by GLS less than 17% is defined as significant deterioration.^9^, ^23^, ^26^ Previous studies indicate that deterioration in GLS, as well as in LS, during and early after anthracycline treatment may identify individuals at higher risk of subsequent cardiac dysfunction.^14^, ^27^, ^28^ In children, the prognostic importance of GLS and LS warrants further clarification and these methods are not included in standard pediatric cancer surveillance protocols.^8^, ^29^ More sensitive and predictive markers of cardiovascular dysfunction would enable identification of patients who could benefit from more frequent surveillance, to enable timely interventions during and after completing cancer treatment with impact on prognosis.^21^, ^30^, ^31^, ^32^

## 2 METHODS

### 2.1 Patient population and study design

In this retrospective, single‐center, population‐based study, we have re‐evaluated cardiac function in serial echocardiograms performed during treatment and clinical follow‐up in childhood sarcoma patients treated according to protocols including high cumulative doxorubicin‐equivalent doses^5^ (≥250 mg/m^2^). The aims of the study were to explore the feasibility of different echocardiographic methods for evaluating left ventricular systolic function (LVEF; FS; GLS; LS), as well as the incidence and timing of systolic dysfunction measured by these methods, during and early after anthracycline treatment.

The study inclusion criteria were age 0–18 years at sarcoma diagnosis and treatment according to protocols including cumulative doxorubicin equivalent doses ≥250 mg/m^2^ at Astrid Lindgren Children's Hospital, Stockholm, during the period 2010–2021. Patients meeting the inclusion criteria were identified through the Swedish Childhood Cancer Epidemiology Group register. Exclusion criteria were age >18 years at cancer diagnosis; cancer diagnosis other than sarcoma; treatment protocol without cumulative doxorubicin equivalent doses ≥250 mg/m^2^; and cardiac disease diagnosed before initiation of cancer therapy.

The patient cohort included 41 patients (14 females and 27 males) diagnosed with sarcoma; 24 with osteosarcoma, 15 with Ewing sarcoma and two patients with soft tissue sarcoma. Median age at the first echocardiogram (at baseline, i.e., after cancer diagnosis but before starting therapy in 40/41 patients) was 12.7 years (range 2.1–17.8 years).

All available echocardiograms were re‐analyzed, and medical reports were reviewed in all included patients. Patient outcomes and echocardiographic variables were longitudinally correlated to pre‐defined intervals of cumulative doxorubicin equivalent doses during treatment (0–59, 60–80, 120–180, 180–225, 240–300, 340–375, and ≥450 mg/m^2^) and to time after the last anthracycline treatment during follow‐up. The dose intervals correspond to the number of administered treatment cycles with anthracyclines; doxorubicin equivalent doses of 60, 75, and 80 mg/m^2^ were administered each treatment cycle for Ewing sarcoma, osteosarcoma and soft tissue sarcoma, respectively. The equivalence ratio of doxorubicin to other anthracyclines according to Feijen et al^5^ was used. Following diagnosis of symptomatic heart failure, subsequent echocardiographic data were excluded in the final analysis. Ethical approval was received from the Swedish Ethical Review Board (Dnr 2020‐02385 and Dnr 2021‐06061‐02).

### 2.2 Echocardiographic image analysis

Echocardiographic datasets, previously acquired during cancer treatment and clinical follow‐up and stored in the archiving system Siemens Syngo Dynamics at the Pediatric Cardiology Unit, Astrid Lindgren Children's Hospital, were re‐analyzed. Left ventricular peak systolic deformation analysis by LS and GLS were performed in accordance with guidelines^33^ using vendor‐neutral software (TOMTEC Corporation). In summary, LS was measured in apical four‐chamber views whereas GLS was automatically calculated from longitudinal strain measurements in apical four‐chamber, apical three‐chamber and apical two‐chamber views. Re‐analysis of echocardiographic data was performed by two senior echocardiographers, supervised by one pediatric cardiologist. Cardiac chamber quantification and functional measurements were performed according to recommendations in current guidelines^17^, ^18^, ^34^ and z‐scores were calculated for chamber dimensions.^35^ When LVEF analysis according to the biplane method of disk summation was not available, visual LVEF (vLVEF) was performed. Visual LVEF was preferred over estimation of LVEF by using the Teicholz formula, as the latter is reported inadequate in children.^17^, ^18^ To avoid confusion, the values of GLS and LS were presented as positive numbers.^36^ Cut‐off values for defining ventricular dysfunction were in accordance with cardio‐oncological guidelines as follows: LVEF <53%, FS <0.28, LS <17%, and GLS <17%.^8^, ^9^, ^10^

### 2.3 Statistical analysis

The association between abnormal LS (<17%) at any time during treatment and vLVEF and FS after the end of treatment was evaluated at follow‐up within 6‐months and during the period of 1–2 years after the last anthracycline treatment. For dichotomised vLVEF and FS values, a 2 × 2 contingency table was set up and one‐sided Fisher's Exact Test was used to evaluate if decrease in vLVEF to values <53% and FS to values <0.28 were positively associated with abnormal LS (<17%) at any time during treatment at the 5% significance level. The null hypothesis was that a decrease in vLVEF to <53% and FS to <0.28 were independent of abnormal LS during treatment. For continuous vLVEF and FS values, the one‐sided Wilcoxon Test was used to test if the median values of vLVEF and FS were lower in the group of patients with abnormal LS during treatment at 5% significance level. The null hypothesis was that there would be no difference in the median values of vLVEF and FS between patients with abnormal LS (<17%) at any time during treatment and those with normal LS (≥17%) throughout treatment.

## 3 RESULTS

### 3.1 Patient characteristics

The patient cohort (Table 1) included 41 patients (14 females and 27 males) diagnosed with sarcoma; osteosarcoma (*n* = 24), Ewing sarcoma (*n* = 15), and soft tissue sarcoma (*n* = 2). The median age at the first echocardiogram was 12.7 years (range 2.1–17.8 years). Patients diagnosed with osteosarcoma and Ewing sarcoma received a median cumulative dose of doxorubicin equivalents of 450 mg/m^2^ (range 225–450 mg/m^2^, administered in cycles of 75 mg/m^2^), and 360 mg/m^2^ (range 300–360 mg/m^2^, administered in cycles of 60 mg/m^2^), respectively. Two patients with soft tissue sarcoma received doses of 240 and 320 mg/m^2^ doxorubicin equivalents (administered in cycles of 80 mg/m^2^). None of the patients received chest irradiation including the heart or the great vessels. The median follow‐up time was 32 months (range 6–97 months) after the first and 26 months (range 0–88 months) after the last anthracycline therapy. At the end of the study period, 30 of the 41 patients (73%) were alive. No patients were diagnosed with renal dysfunction (Glomerular Filtration Rate <60 mL/min/1.73 m^2^) or arterial hypertension, and there were no cardiac deaths during the entire study period. One patient with osteosarcoma was diagnosed with symptomatic heart failure of NYHA class III, 4.5 months after cessation of therapy with a cumulative doxorubicin dose of 450 mg/m^2^. Echocardiography at the time of clinical diagnosis of heart failure, revealed an LVEF of 15% (by the biplane method of disks and by visual evaluation), FS of 0.10 and LS of 5%. All the subsequent echocardiograms for this patient were excluded from the final analysis. At the presentation of heart failure, the biomarker N‐Terminal Pro–Brain Natriuretic Peptide was moderately elevated, 7900 ng/L (normal reference value <160 ng/L), but high sensitivity cardiac Troponin‐T was normal. Prior to the presentation of symptomatic heart failure, six normal echocardiograms were performed during treatment and one echocardiogram 1 month after ending treatment. The latter, showed a normal FS (0.30) and a slightly reduced visual LVEF (50%). Unfortunately, neither LS nor biplane LVEF was available at this timepoint, due to poor quality of the stored echocardiographic images from apical views. No preceding risk factors such as arrhythmia, septicaemia, anemia, arterial hypertension, electrolyte disturbances, or renal failure were identified. With an LVEDD of +2.2 z‐scores, this was the only patient with significant dilatation of the left ventricle (LVEDD ≥ +2.0 z‐scores). A maximum LVEDD of +2.4 z‐scores was seen before improvement of cardiac systolic function occurred. Cardiac systolic function recovered completely (LVEF >53%, FS >0.28, LS >17%) with standard heart failure treatment^37^ within 3 months. The patient succumbed to relapsed osteosarcoma 1 year and 3 months after the cessation of anthracycline therapy. This patient with symptomatic heart failure was the only patient in the cohort who received heart failure medication.

**TABLE 1:** Characteristics of the patient cohort.

Diagnosis	All patients, *n* = 41			
	All diagnoses	Osteosarcoma	Ewing sarcoma	Soft tissue sarcoma
All patients, *n* (%)	41 (100)	24 (59)	15 (36)	2 (5)
Females, *n* (%)	14 (34)	7 (29)	7 (47)	0 (0)
Males, *n* (%)	27 (66)	17 (71)	8 (53)	2 (100)
Age in years at baseline echocardiogram, median (range)	12.7 (2.1–17.8)	12.6 (6.7–17.6)	12.3 (2.1–17.8)	14.8 (13.4–16.3)
Total cumulative doxorubicin equivalent dose^a^ mg/m^2^, median (range)	375 (225–450)	450 (225–450)	360 (300–360)	280 (240–320)
Follow up in months after start of treatment, median (range)	32.0 (6–97)	26.5 (6–97)	47.0 (8–69)	25.5 (15–36)
Follow up in months after end of treatment, median (range)	26.0 (0–88)	14.8 (0–88)	44.0 (5–65)	21.5 (12‐31)
Survivors at last follow‐up, *n* (%)	30/41 (73%)	17/24 (71%)	11/15 (73%)	2/2 (100%)
Hypertension (>95 quartile) during the study period, *n* (%)	0 (0%)	0 (0%)	0 (0%)	0 (0%)
Renal failure, (GFR <60 mL/min/1.73 m^2^), during the study period, *n* (%)	0 (0%)	0 (0%)	0 (0%)	0 (0%)
Symptomatic heart failure^b^, during the study period, *n* (%)	1 (2%)	1 (4%)	0 (0%)	0 (0%)

Abbreviations: GFR, glomerular filtration rate; N, numbers.

^a^ Doxorubicin equivalent doses according to Feijen et al., 2019.

^b^ Symptomatic heart failure defined as NYHA II‐IV.

### 3.2 Feasibility of retrospective analysis of echocardiograms

**FIGURE 1:**
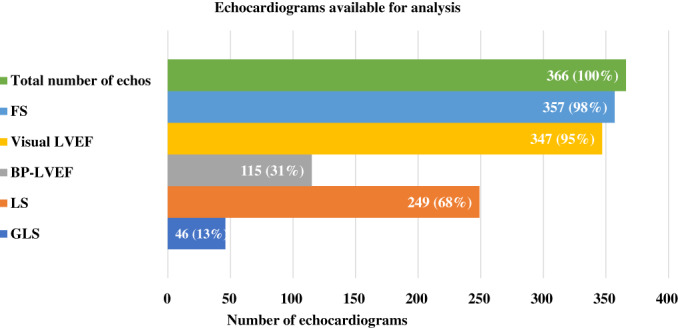
Echocardiograms with available data for analyses in accordance with guidelines. BP‐LVEF, biplane left ventricular ejection fraction; Echo, echocardiograms; FS, fractional shortening; GLS, global longitudinal strain; LS, longitudinal strain; visual LVE, visual left‐ventricular ejection fraction.

In total, 366 echocardiograms were available for re‐analysis (Figure 1). The extent of acquired data and image quality, especially in echocardiograms performed earlier than 2015, were limited. Analyses in accordance with guidelines^18^, ^33^ could only be performed in a subset of echocardiograms: 46/366 (13%) for GLS, 249/366 (68%) for LS, 115/366 (31%) for LVEF by the biplane method of disk summation, 347/366 (95%) for vLVEF and 357/366 (98%) for FS. Evaluation of FS and vLVEF were eligible in all echocardiograms that were feasible for LS. Eligibility of LVEF estimation by the biplane method of disk summation was hampered by inadequate image quality including foreshortening of two chamber views.^18^ GLS assessment was restricted by the lack of all three necessary echocardiographic views.^33^ The main reason for LS ineligibility in our retrospective material was restricted image sectors from the apical four chamber view (the myocardium was not visualized throughout the cardiac cycle).

### 3.3 Echocardiographic results

Of the 249 echocardiograms eligible for LS analysis (Table 2), abnormal values were recorded for LS (<17%) in 105 (42%), for FS (<0.28) in 12 (5%) and for LVEF (<53%) in 30 cases (12%).

**TABLE 2:** Re‐analysis of FS and LVEF in echocardiograms eligible for LS‐analysis.

Echocardiograms eligible for LS		Normal FS (>0.28) + Normal vLVEF (≥53%)	Normal FS (>0.28) + Abnormal vLVEF (<53%)	Abnormal FS (<0.28) + Normal vLVEF (≥53%)	Abnormal FS (<0.28) + Abnormal vLVEF (<53%)
Total *n* (%)	249 (100)				
Abnormal LS (<17%) *n* (%)	105 (100)	75 (71)	19 (18)	3 (3)	8 (8)
Normal LS (≥17%) *n* (%)	144 (100)	0 (0)	3^a^ (2)	1^b^ (1)	0 (0)

Abbreviations: FS, fractional shortening; LS, longitudinal strain; N, numbers; vLVEF, visual Left ventricular ejection fraction.

^a^ LVEF 50%, in all three.

^b^ FS 0.27.

LS was the only affected parameter, that is, FS and vLVEF were concomitantly normal in 75/105 cases (71%). Besides LS, only one other systolic parameter (FS or vLVEF) was concomitantly affected in 22/105 (21%), while both FS and vLVEF were co‐affected in 8/105 (8%).

Normal LS (≥17%), was registered in 144 echocardiograms (48%). In these echocardiograms, borderline values of FS (0.27) were detected in three and borderline vLVEF (50%) in one case, but there were none with abnormal values in both vLVEF and FS. The results of FS and vLVEF measurements were contradictory in 35 of all 366 echocardiograms. LS analysis was feasible in 22 of these 35 echocardiograms.

**FIGURE 2:**
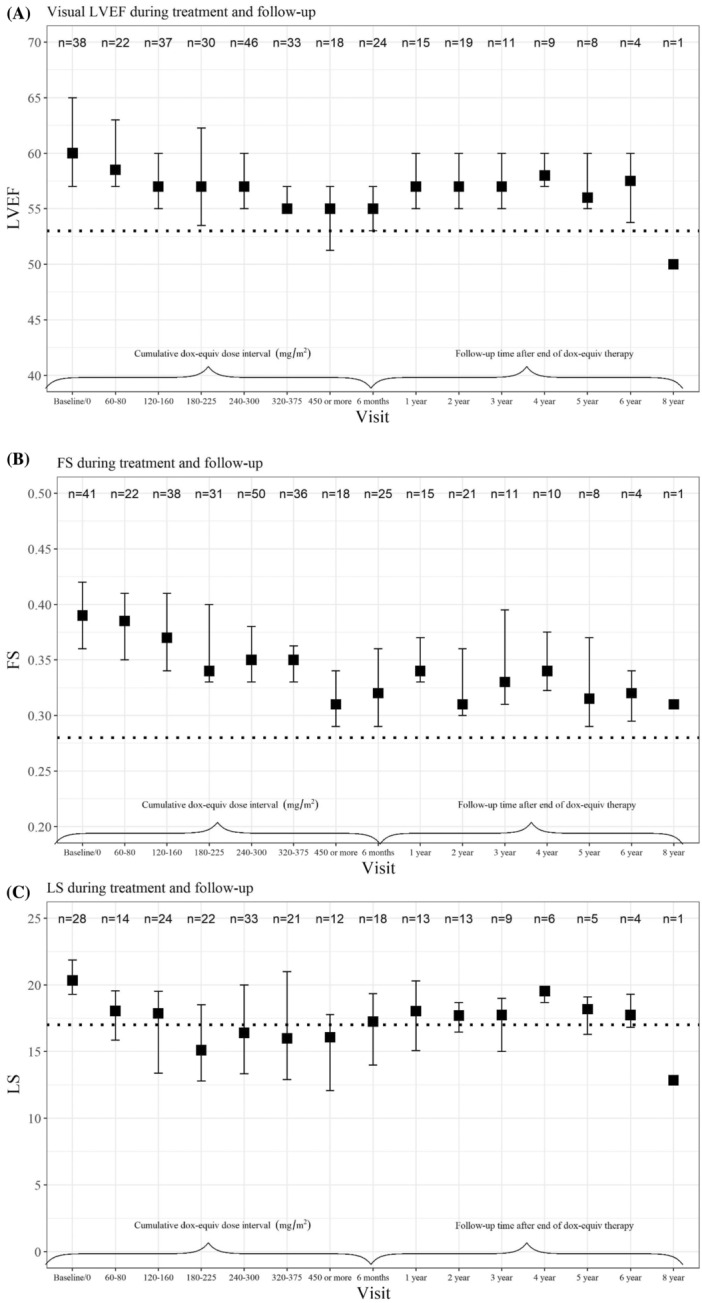
(A), (B), and (C) Longitudinal follow‐up of median values and interquartile range, for vLVEF, FS, and LS respectively, according to cumulative doxorubicin equivalent dose‐intervals (mg/m2) during treatment and time after last anthracycline dose during follow‐up. Dox‐equiv, doxorubicin equivalent; FS, fractional shortening; LS, longitudinal strain; LVEF, left ventricular ejection fraction.

The median values of the echocardiographic systolic parameters with corresponding interquartile range at different cumulative doxorubicin equivalent dosage‐intervals during treatment and at different timepoints during follow‐up were calculated. For vLVEF and FS, the median values remained above the thresholds of normal (vLVEF 53% and FS 0.28 respectively) during and after treatment. The median values for LS were abnormal (<17%) from cumulative doxorubicin equivalent doses of 180–225 mg/m^2^, but returned to normal values (≥17%) at 6 months follow‐up after ending treatment (Figure 2A–C).

**FIGURE 3:**
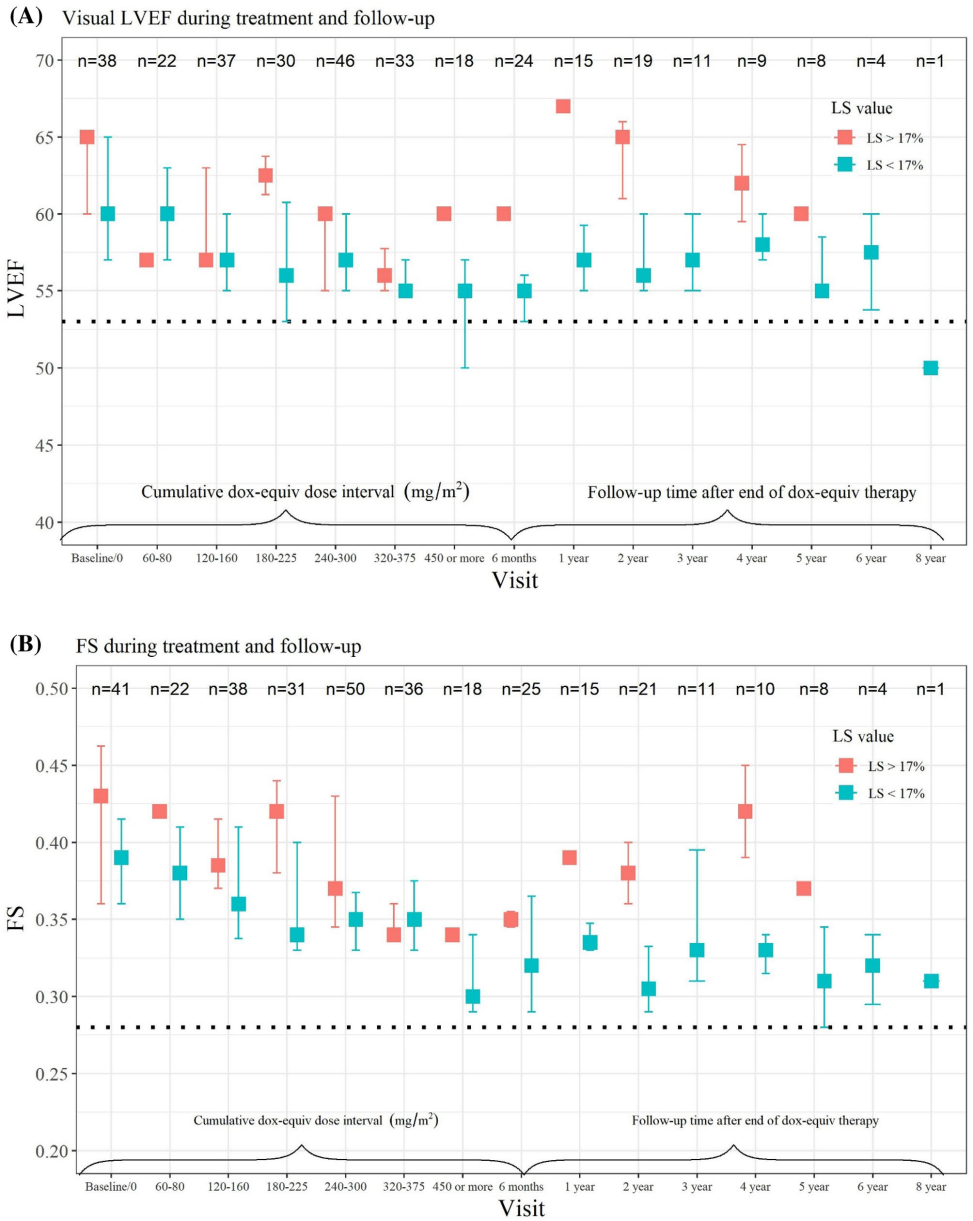
(A) and (B) Longitudinal follow‐ups of median values and interquartile range of visual LVEF and FS in patients with normal LS (≥17%) at all times (red) or abnormal LS (<17%) (green) at any time, according to cumulative doxorubicin equivalent dose‐intervals (mg/m^2^) during treatment and time after last anthracycline dose during follow‐up. Dox‐equiv, doxorubicin equivalent; FS, fractional shortening; LS, longitudinal strain; LVEF, left ventricular ejection fraction.

In a sub‐analysis, the cohort was dichotomised into patients with at least one echocardiogram with abnormal LS (<17%) and patients with normal LS (≥17%) throughout treatment (Figure 3A,B). In the sub‐group with abnormal LS, the median values of vLVEF and FS were lower than in the sub‐group with normal LS throughout treatment. The question whether patients with abnormal LS during treatment demonstrated lower median values of vLVEF or FS at follow‐up within 6 months, as well as during the period 1–2 years after ending treatment, could not be answered due to limited numbers of available echocardiograms. The Fisher's Exact Test was used to evaluate if significant deterioration in vLVEF (<53%) and FS (<0.28) were positively associated with abnormal LS (<17%) during treatment at 5% significance level. For continuous vLVEF and FS values, one‐sided Wilcoxon Test was used. Both tests demonstrated statistically non‐significant results (Table S1). We acknowledge that the Wilcoxon tests were used on groups that are highly unbalanced, which affected the power of the tests.

### 3.4 Image quality

The relationship between frame rate and heart rate (FR/HR‐ratio) as a measure for image quality may interfere with strain analysis.^38^ We compared the FR/HR‐ratio in echocardiograms analyzed for LS (Figures S1A,B). The FR/HR‐ratio was ≥0.7 in 155/249 echocardiograms (62%). In the 105 echocardiograms with LS <17% the FR/HR‐ratio was ≥0.7 in 59/105 (56%), whereas in the 144 eligible echocardiograms with unimpaired LS, the FR/HR‐ratio was ≥0.7 in 96/144 (67%).

## 4 DISCUSSION

Cardiac dysfunction before, during and after cancer treatment has important impact on both cancer and overall outcome, as well as long‐term quality of life, hence cardiotoxic cancer treatment regimens require serial evaluation of cardiac function by echocardiography.^8^, ^9^, ^10^, ^39^ Early detection of cardiac dysfunction, prior to overt symptomatic heart failure, is considered vital to enable timely interventions.^7^, ^16^, ^39^ The standard echocardiographic methods for longitudinal surveillance of cardiac function in children, LVEF, and FS,^17^, ^18^ exhibit limitations regarding reproducibility as well as sensitivity to detect small changes in left ventricular systolic function.^19^, ^40^ The limited sensitivity is problematic, since significant reduction in LVEF as well as in and FS, often occur late in the process of cancer therapy induced cardiac dysfunction.^25^ Limited reproducibility poses a challenge already during cancer treatment since reduction of LVEF or FS may motivate treatment alterations that may interfere with long‐term cancer outcome. In conclusion, more sensitive and reproducible surveillance methods are warranted.

In adults, myocardial deformation, measured as GLS by 2D speckle‐tracking echocardiography, has become the preferred parameter for early detection of asymptomatic cancer therapy‐related cardiac dysfunction due to its high reproducibility and sensitivity,^9^, ^10^, ^11^ even if the prognostic impact is yet to be proven. The availability of GLS measurements in children, especially during cancer treatment, might be hampered by the need of a high‐quality images from three apical echocardiographic views. Including analysis of LS has therefore been suggested as an option when GLS is not feasible,^27^, ^28^ since LS‐analysis only requires a standard apical four‐chamber view of the left ventricle of adequate image quality and frame rate.^41^, ^42^ The feasibility, as well as the reproducibility, sensitivity and specificity, of an echocardiographic method are of relevance both in research and in clinical practice. In our retrospective material, LS demonstrated a higher feasibility (68%) than GLS (13%) as well as LVEF measured by the recommended biplane method (31%).^8^, ^18^ This may, in part, reflect the limitations of a retrospective dataset including previously acquired echocardiographic images. However, it may also be ascribed to the differences in feasibility between the echocardiographic methods that may be of relevance in clinical practice as well as in research.

A good correlation between GLS and LS measurements has been demonstrated by others,^43^ but GLS is recommended, when available, due to higher reproducibility.^10^

Longitudinal strain was the most frequently affected parameter of systolic function (impaired in 42%, as compared to impaired FS in 5% and vLVEF in 12%). This finding, together with results from previous studies of childhood cancer survivors, indicates a superior sensitivity of LS compared to LVEF and FS.^27^, ^28^ Median values for vLVEF and FS remained above the cut‐off values for defining ventricular dysfunction (vLVEF <53% and FS <0.28 respectively) during and after treatment. The median values for LS were abnormal (<17%) from cumulative doxorubicin equivalent doses of 180–225 mg/m^2^, indicating myocardial injury at lower doses than considered to be “high risk”.^10^ This finding may indicate a dose–response relation. Median values for LS remained abnormal during further treatment, but returned to normal values (≥17%) within 6 months follow‐up after ending treatment. The correlation between LS impairment during treatment and subsequent cardiac dysfunction measured by LS, vLVEF, or FS, hence, the sensitivity and specificity of LS as a prognostic marker for subsequent cardiac dysfunction, could not be addressed in our cohort due to limited cohort size and follow‐up time. However, a recent study indicates a role for LS as a risk marker for subsequent heart failure in childhood cancer survivors.^27^

The methodological limitations of standard echocardiographic surveillance of pediatric cancer patients, including only FS and LVEF, are demonstrated in this study. Although FS had excellent feasibility, as available in 98% (357/366) of the echocardiograms, there was only one case with impaired FS (0.27), as the only affected systolic parameter. In contrast, LVEF measurements by the recommended biplane method of disk summation^18^ were feasible in only 31% of the cases, due to inadequate image quality including foreshortening of two chamber views. Visual LVEF was on the other hand assessable in 95% (347/366) of all echocardiograms. High reproducibility of vLVEF depends on reader experience and can hence not be generalized.^44^ The results of FS and LVEF measurements were contradictory in 35/366 echocardiograms, which might pose a problem in clinical decision‐making. In these cases, GLS or LS measurements may facilitate the evaluation of left ventricular systolic performance.

Besides the size of the patient cohort and sort follow‐up time, a weakness of this study, shared by other retrospective studies on echocardiograms performed in clinical practice, was the limitation of acquired images.^28^, ^45^

## 5 CONCLUSIONS

Despite the limitations of cohort size and follow‐up time, our results demonstrate a high feasibility of LS also in retrospective echocardiographic datasets. Our results indicate that LS entails higher sensitivity for detecting subclinical changes in cardiac function compared to FS and LVEF. In conjunction with previous studies,^27^, ^28^ this study contributes to strengthening the proposal of including LS (when GLS is unavailable) in standard echocardiographic surveillance protocols for longitudinal monitoring of children during and after anthracycline treatment. LS, and preferably GLS, may be particularly useful to further scrutinize the significance of borderline FS or LVEF values and aid in decision‐making regarding surveillance intensity, both during and after anthracycline treatment.^10^, ^27^, ^28^, ^41^

To improve the understanding of the variable course of individual patients and to provide outcome measures for further research on early detection of cardiovascular toxicity, adherence to detailed standardized echocardiographic protocols including left ventricular systolic strain analysis^46^ (preferably GLS, alternatively LS) should be implemented in clinical practice. We are now preparing a prospective study to validate LS and GLS in echocardiographic surveillance during and after anthracycline treatment in children to confirm and expand the retrospective results presented here.

## AUTHOR CONTRIBUTIONS

All authors had full access to the data in the study and take responsibility for the integrity of the data and the accuracy of the data analysis. *Conceptualization*: Maria Sjöborg Alpman, Agneta Månsson Broberg, Nikolas Herold, Aristomenis Manouras, and Jan‐Inge Henter. *Methodology*: Maria Sjöborg Alpman, Agneta Månsson Broberg, Nikolas Herold, and Jan‐Inge Henter. *Validation*: Maria Sjöborg Alpman, Annica Jarting, and Kerstin Magnusson. *Investigation*: Maria Sjöborg Alpman, Annica Jarting, and Kerstin Magnusson. *Formal Analysis*: Maria Sjöborg Alpman, Agneta Månsson Broberg, and Nikolas Herold. *Resources*: Jan‐Inge Henter, Agneta Månsson Broberg, and Nikolas Herold. *Writing—Original Draft*: Maria Sjöborg Alpman, Agneta Månsson Broberg, and Nikolas Herold. *Writing—Review and Editing*: Maria Sjöborg Alpman, Agneta Månsson Broberg, Nikolas Herold, Aristomenis Manouras, Jan‐Inge Henter, Annica Jarting, and Kerstin Magnusson. *Visualization*: Maria Sjöborg Alpman. *Supervision*: Jan‐Inge Henter. *Funding Acquisition*: Jan‐Inge Henter.

## CONFLICT OF INTEREST STATEMENT

The authors have stated explicitly that there are no conflicts of interest in connection with this article.

## ETHICS STATEMENT

Ethical approval was received from the Swedish Ethical Review Board (Dnr 2020‐02385 and Dnr 2021‐06061‐02).

## Supporting information


**Table S1.** Longitudinal echocardiographic characteristics of patients with abnormal LS (<17%) at any time during anthracycline treatment and normal LS (≥17%) at all times during treatment, with respect to LS, vLVEF and FS during follow‐up within 6 months and at 1–2 years after end of anthracycline treatment. Statistical analysis was performed to evaluate the association between abnormal LS (<17%) at any time during treatment and vLVEF and FS at follow‐up within 6 months and during the period 1–2 years after the last anthracycline treatment. Fisher's exact test and Wilcoxon test returned statistically non‐significant results. FS, fractional shortening; IQR, interquartile range; LS, longitudinal strain; n, numbers; NA, not available; vLVEF, visual left ventricular ejection fraction; yr, year.
**Figure S1.** (A) and (B) Echocardiograms with normal LS (≥17%) as well as echocardiograms with abnormal LS (<17%) demonstrate a FR/HR‐ratio ≥0.7 in a majority of cases: FR, frame rate; HR, heart rate; LS, longitudinal strain.

## Data Availability

The data supporting the findings of this study are available upon request from the corresponding author. The data are not publicly available due to privacy or ethical restrictions.
