# Supplementary material for: Longitudinal strain analysis for assessment of early cardiotoxicity during anthracycline treatment in childhood sarcoma: A single center experience
Source: Cancer Rep (Hoboken). 2023 Jun 24;6(9):e1852. doi: 10.1002/cnr2.1852 (PMC10480418; doi:10.1002/cnr2.1852)
Supplement: Supplementary file 1 — Table S1. Longitudinal echocardiographic characteristics of patients with abnormal LS (<17%) at any time during anthracycline treatment and normal LS (≥17%) at all times during treatment, with respect to LS, vLVEF and FS during follow‐up within 6 months and at 1–2 years after end of anthracycline treatment. Statistical analysis was performed to evaluate the association between abnormal LS (<17%) at any time during treatment and vLVEF and FS at follow‐up within 6 months and during the period 1–2 years after the last anthracycline treatment. Fisher's exact test and Wilcoxon test returned statistically non‐significant results. FS, fractional shortening; IQR, interquartile range; LS, longitudinal strain; n, numbers; NA, not available; vLVEF, visual left ventricular ejection fraction; yr, year. Figure S1. (A) and (B) Echocardiograms with normal LS (≥17%) as well as echocardiograms with abnormal LS (<17%) demonstrate a FR/HR‐ratio ≥0.7 in a majority of cases: FR, frame rate; HR, heart rate; LS, longitudinal strain. [file CNR2-6-e1852-s001.docx]

## Supporting information

Supplemental TABLE S1 Longitudinal echocardiographic characteristics of patients with abnormal LS (<17%) at any time during anthracycline treatment and normal LS (≥17%) at all times during treatment, with respect to LS, vLVEF and FS during follow-up within 6 months and at 1-2 years after end of anthracycline treatment.

Statistical analysis was performed to evaluate the association between abnormal LS (<17%) at any time during treatment and vLVEF and FS at follow up within 6 months; and during the period 1-2 years after the last anthracycline treatment. Fisher Exact test and Wilcoxon test returned with statistically non-significant results.

|  | Level | Overall | LS <17% during treatment | LS ≥17%  during treatment | Fisher Exact Test | Wilcoxon Test |
| --- | --- | --- | --- | --- | --- | --- |
| n |  | 41 | 30 | 8 |  |  |
| LS <17%  within 6months;  n (%) | **1** | 6 (14.6) | 5 (16.7) | 1 (12.5) |  |  |
|  | **0** | 6 (14.6) | 4 (13.3) | 2 (25.0) |  |  |
|  | **NA** | 29 (70.7) | 21 (70.0) | 5 (62.5) |  |  |
| LS <17%  1yr-2yr;  n (%) | **1** | 9 (22.0) | 7 (23.3) | 2 (25.0) |  |  |
|  | **0** | 5 (12.2) | 3 (10.0) | 1 (12.5) |  |  |
|  | **NA** | 27 (65.9) | 20 (66.7) | 5 (62.5) |  |  |
| LVEF <53%  during treatment; n (%) | **1** | 12 (29.3) | 10 (33.3) | 2 (25.0) |  |  |
|  | **0** | 27 (65.9) | 20 (66.7) | 5 (62.5) |  |  |
|  | **NA** | 2 (4.9) | 0 (0.0) | 1 (12.5) |  |  |
| LVEF <53%  within 6months; n (%) | **1** | 3 (7.3) | 3 (10.0) | 0 (0.0) | 1.00 |  |
|  | **0** | 13 (31.7) | 10 (33.3) | 3 (37.5) |  |  |
|  | **NA** | 25 (61.0) | 17 (56.7) | 5 (62.5) |  |  |
| LVEF <53%  1yr-2yr; n (%) | **1** | 3 (7.3) | 2 (6.7) | 1 (12.5) | 0.51 |  |
|  | **0** | 18 (43.9) | 14 (46.7) | 3 (37.5) |  |  |
|  | **NA** | 20 (48.8) | 14 (46.7) | 4 (50.0) |  |  |
| FS <0.28  during treatment; n (%) | **1** | 7 (17.1) | 7 (23.3) | 0 (0.0) |  |  |
|  | **0** | 34 (82.9) | 23 (76.7) | 8 (100.0) |  |  |
| FS <0.28  within 6months; n (%) | **1** | 3 (7.3) | 3 (10.0) | 0 (0.0) | 0.53 |  |
|  | **0** | 14 (34.1) | 9 (30.0) | 4 (50.0) |  |  |
|  | **NA** | 24 (58.5) | 18 (60.0) | 4 (50.0) |  |  |
| FS <0.28  1yr-2yr; n (%) | **1** | 1 (2.4) | 1 (3.3) | 0 (0.0) | 1.00 |  |
|  | **0** | 21 (51.2) | 15 (50.0) | 4 (50.0) |  |  |
|  | **NA** | 19 (46.3) | 14 (46.7) | 4 (50.0) |  |  |
| LVEF  within 6months; median [IQR] |  | 55.00  [54.50, 57.00] | 55.00 [53.00, 55.00] | 57.00[56.00, 58.50] |  | 0.14 |
| LVEF  1yr-2yr;  median [IQR] |  | 57.00 [55.00, 60.00] | 56.00 [55.00, 57.75] | 58.50 [55.25, 61.25] |  | 0.47 |
| FS within 6months;  median [IQR] |  | 0.32 [0.29, 0.37] | 0.30 [0.28, 0.33] | 0.37 [0.36, 0.38] |  | 0.06 |
| FS 1yr-2yr; median [IQR] |  | 0.31 [0.29, 0.37] | 0.30 [0.29, 0.33] | 0.38 [0.36, 0.38] |  | 0.09 |

LS, Longitudinal Strain; vLVEF, Visual Left Ventricular Ejection Fraction; FS, Fractional Shortening; n, numbers; NA, not available; IQR, interquartile range; yr, year.

Supplemental FIGURES S1

A

B

Supplemental FIGURES S1A and S1B Echocardiograms with normal LS (≥17%) as well as echocardiograms with abnormal LS (<17%) demonstrate a FR/HR-ratio ≥0.7 in a majority of cases: FR, Frame Rate; HR, Heart Rate; LS, Longitudinal Strain
